# Plummer on Adipose Tumors

**Published:** 1848

**Authors:** John T. Plummer

**Affiliations:** Richmond, Indiana


### ARTICLE V.

*Adipose Tumors.* By JOHN T. PLUMMER, M.D., of Richmond, Indiana.

As Medical Journals are often made the store-houses of facts not immediately appropriated, I take the liberty of asking a place for a case which, although of little importance in itself, may be of use in a theoretical bearing.

"Steatomatous tumors," says Astley Cooper, "are generally said to be encysted, but it is not so. The character of them is lobulated, consisting of lobes united together by cellular membrane." Again: "They are quite unattended with pain." And, finally: "They are the growth of the adipose cells."

If the distinguished surgeon just quoted, intended to say that steatomes are in themselves insensible, he has been unhappy in the use of his language; if he means that these tumors do not excite pain—that is, "are quite unattended with pain"—I must be allowed to say, that my experience does not confirm the unqualified statement. Indeed, most of the applications made to me in these cases, have arisen from the very fact that the tumors occasioned pain; and sometimes, too, when the tumors were comparatively small. One, I find in my notes, not more than one and a half inch diameter, on the top of the foot, created severe pain, even through to the sole of the foot.

Neither have I always observed the lobulated character mentioned; and especially was it absent in the following case:

A woman, between 60 and 70 years of age, applied to me on account of a tumor which lay above the left clavicle and extended up the neck. It had existed about eight years and was now so painful that she was desirous of having it removed. It was of an ovoid form, and, from its mobility, I had no doubt of its being what we generally call an encysted tumor; although we do not always find them enclosed in a *cyst*. I had removed, a month or two previously, from beneath the scalp of the same patient, a honeyed wen, (melliceris,) and a mealy wen, (atheroma,) by evacuating the sacs, and blowing into their cavities a small portion of red oxide of mercury, and she was desirous of having the same method applied to the present tumor. The size of the wen being the only objection to gratifying her in this particular, I punctured the mass; but not being able to press out its substance, I made an incision nearly the whole length of the tumor, and speedily dissected it out. It proved to be a steatome, (improperly, though conveniently, ranked among encysted tumors) of very firm consistence, and, in my experience and recollection of authors, of an unusual structure.

In the centre of the mass, which was four or five inches long, was a nucleus about two inches in diameter; and around this lay a succession of strata, like the coats of an onion, varying from one-eighth to one-fourth of an inch in thickness, and completing the tumor. These layers were slightly united to one another, and to the nucleus by cellular tissue. As far as the eye could perceive, the whole structure of the tumor was homogenous. When thrown into water, the tumor sank to the bottom, but quickly exuded some of its oily portions, which rose to the surface in rich yellow dots.

If this tumor originated from the "morbid growth of the adipose cells," by what peculiar action were those cells disposed in this stratified form?

This question in the hands of such morphologists as Goethe, Mohl, and Henfrey, who, out of a simple cell, can construct not only the meanest plant, but also the stateliest complicated oak, would, perhaps, not be of difficult solution; but my province, in the present case, is simply to record appearances.

